# A Multicenter Physician Survey Evaluating the Use of Ki-67 in Breast Cancer Management in Canada

**DOI:** 10.3390/biomedicines12112471

**Published:** 2024-10-28

**Authors:** Jennifer Leigh, Sharon F. McGee, Lisa Vandermeer, Phillip Williams, Moira Rushton

**Affiliations:** 1 Division of Medical Oncology, Department of Medicine, The Ottawa Hospital, Ottawa, ON K1H 8L6, Canadamoirushton@toh.ca (M.R.); 2 Cancer Therapeutics Program, Ottawa Hospital Research Institute, Ottawa, ON K1H 8L6, Canada; 3 Department of Pathology, The Ottawa Hospital, Ottawa, ON K1Y 4E9, Canada

**Keywords:** breast cancer, Ki-67, endocrine response, short-course ET

## Abstract

Background: Ki-67’s response to pre-operative endocrine therapy (ET) in early breast cancer is an evidence-based tool to guide adjuvant treatment decisions. Physicians across Canada were surveyed to explore current practice patterns and perceived barriers to the use of Ki-67 in practice. Methods: Physicians were invited to participate in an anonymous survey and were eligible if they prescribed systemic therapy for breast cancer in Canada. Respondents were asked to describe their usage of Ki-67, perceptions of the evidence surrounding Ki-67 ET response, and interest in future trials using this approach. Results: The survey received 48/163 responses (29.4%). The majority of respondents (97.6%) reported access to Ki-67 testing upon request. Treatment decisions for adjuvant Abemaciclib was the most common reason (97.6%), followed by adjuvant chemotherapy decisions (16.7%). Only 19.0% had used Ki-67’s response to pre-operative ET in practice. Common barriers to this approach that were identified included a lack of awareness from other providers (54.8%), an increased resource requirement (54.8%), and a lack of timely medical oncology consultation (52.4%). The majority of physicians (85.3%) reported that they would participate in future trials using the Ki-67 endocrine response, and that rate of treatment decision change (95.2%) and cost analysis (42.3%) were important endpoints. Conclusions: Despite the widespread availability of Ki-67 testing, few physicians in Canada currently use it to assess endocrine response, predominantly due to logistical and resource constraints. There is a high level of interest in participating in future trials using this strategy, which should focus on disease related outcomes, feasibility, and the financial impact on the public healthcare system.

## 1. Introduction

Estrogen receptor-positive (ER-positive) and HER-2-negative tumors make up the largest group of all breast cancer (BC) subtypes in North America [1]. In early-stage BC (I-III), adjuvant endocrine therapy (ET) and chemotherapy have demonstrated survival benefits [2]. Traditionally, tumor size and grade and lymph node involvement have guided treatment decisions. Not all patients, however, will benefit from chemotherapy; thus, there has been an effort to identify patients that are highly endocrine sensitive, in whom chemotherapy can be safely omitted, thus avoiding the overtreatment of some BC patients. There are numerous proprietary tests available to clinicians to help identify which patients can avoid chemotherapy, such as OncotypeDx, MammaPrint, and Endopredict, as well as clinical risk scores when incorporating Ki-67—a marker of cell proliferation [3,4,5,6,7]. Higher values have been associated with higher risk of recurrence in early breast cancer, and thus there has been a push to identify whether it can be utilized to assess treatment response and guide adjuvant treatment decision making [8]. Guidelines for Ki-67 testing, however, are heterogeneous, with the European Society for Medical Oncology (ESMO) currently recommending Ki-67 be tested in patients with early-stage BC, whereas the Canadian Association of Pathologists Task Force recommends it be tested only upon medical oncology request [9,10]

Pre-operative endocrine therapy and measurement of endocrine responsiveness through serial Ki-67 testing is another strategy to guide systemic therapy decisions for early-stage hormone-positive breast cancer. The first largescale study to examine this was the POETIC trial, which found that baseline Ki-67 and Ki-67 response to pre-operative endocrine therapy were prognostic in post-menopausal women [11]. They found that patients whose Ki-67 started and remained low (<10%) had the lowest risk of recurrence. Building on this concept, the WSG-ADAPT HR+/HER2- trial looked at whether endocrine sensitivity was predictive for treatment benefit and could be used to guide adjuvant treatment decision making [12,13]. They found that, in pre-menopausal patients, there was no significant difference in 5-year disease-free survival (DFS) between patients with an Oncotype Dx Recurrence Score (RS) of 0–11 and those with an RS of 12–25 who had an endocrine response to pre-operative endocrine therapy. There was, however, a difference between those who did have an endocrine response and those who did not. Taken together, these results highlight the utility of Ki-67 response in prognostication and as a tool for adjuvant therapy decision making, especially in those 50 years or younger. This is particularly important, as the current standard of care for any pre-menopausal female with node-positive disease is adjuvant chemotherapy followed by endocrine therapy [5].

In Canada, each province is responsible for its own oversight and delivery of cancer care. Patients may receive their care at either academic or community hospitals, with the distribution varying by province. Endocrine therapy for the treatment of breast cancer is predominantly managed by medical oncologists who work out of these hospitals. To our knowledge, despite the available evidence, short-course NA endocrine therapy and endocrine response assessment are not strategies routinely used in Canadian cancer centers. The purpose of our study was to survey breast cancer specialists across Canada to understand the current use of Ki-67 testing, knowledge of endocrine response assessments and future interest in this approach to systemic therapy decisions for early-stage BC.

## 2. Materials and Methods

### 2.1. Survey Development

This study is reported in accordance with the CHERRIES statement [14]. The survey was developed by a team of physicians who treat breast cancer and have expertise in prior survey research [15,16,17]. The intended target group was Canadian clinicians who treat breast cancer, including medical oncologists, surgical oncologists, and general practitioners in oncology. The first section of the survey assessed respondent eligibility and collected pertinent demographic data, including location and setting of practice, and years in practice (4 items). The second section collected information on the respondents’ access to and usage of Ki-67 testing (3 items). The third section assessed respondents’ knowledge of the literature surrounding Ki-67 responsiveness and their experience and perception of its uses in clinical practice (6 items). Adaptive questioning was used in this section. Finally, the fourth section presented respondents with a schematic of a potential clinical trial using pre-operative endocrine therapy and Ki-67 responsiveness to guide adjuvant treatment decisions, and it assessed their interest in and perceived barriers to such a study (3 items). The full survey can be found in the Supplementary Materials.

### 2.2. Survey Implementation

Potentially eligible physicians were identified through a publicly available list of physician email addresses that has been used in prior similar survey studies [15]. Technical functionality was tested by study team members prior to sending the survey out. Physicians were considered eligible if they prescribed systemic therapy, practiced in Canada, and were able to complete the survey in English. They were excluded if they were currently practicing outside of Canada and if they had not treated breast cancer in the last three years. The survey was run using Microsoft Forms on a secure account accessible only to the research coordinator. Physicians were sent an invitation to complete the survey, an information sheet outlining the intent of the survey, and a link to complete it electronically. It was initially emailed out on 10 July 2023 and remained open until 30 November 2023. Two reminder emails to complete the survey were sent after initial delivery. Consent was implied from the completion of the survey. Ethics approval was obtained from the Ottawa Health Sciences Network Research Ethics Board (Protocol # 20230298-01H).

### 2.3. Data Analysis

Data were summarized descriptively and analyzed using Microsoft Excel (Version 16.66.1). Responses are reported with the frequency of each answer as a proportion of the total number of respondents for that question. Only complete questionnaires were analyzed. Unique IP addresses were not used to track duplicate survey completion.

## 3. Results

### 3.1. Physician Demographics

The survey was sent to 163 physicians, and we received 48 responses (29.4%). A total of 30 out-of-office replies were received: 12 in the first week and 18 in the second week. There were 42 (87.5%) physicians who reported prescribing systemic therapy for breast cancer. The other six reported not prescribing systemic therapy and thus were considered ineligible. The majority of the eligible respondents worked at an academic center (*n* = 37/42, 88.1%). Length of time in independent practice varied, with 7/42 (16.7%) in practice for less than 5 years, 10/42 (23.8%) 5–10 years, 13/42 (30.9%) 10–20 years, and 12/42 (28.6%) in practice for more than 20 years. The majority of physicians practiced in Ontario (*n* = 26/42, 61.9%), followed by Saskatchewan (*n* = 4/42, 9.52%), Quebec (*n* = 3/42, 7.145), Manitoba (*n* = 3/42, 7.14%), Nova Scotia (*n* = 3/42, 7.14%), Alberta (*n* = 2/42, 4.76%), and British Columbia (*n* = 1/42, 2.38%). Details on physician demographics can be found in Table 1.

### 3.2. Use of Ki-67 Testing

Physicians were asked about their access to Ki-67 testing and its use in practice. Almost all respondents (*n* = 41/42, 97.6%) reported that Ki-67 testing was available for early-stage breast cancer upon request only, and one respondent (2.38%) reported that it was performed reflexively. When asked what they used Ki-67 for in their practice, 41/42 (97.6%) reported using it to guide the use of adjuvant therapies such as Abemaciclib (during the survey period, Ki-67 > 20% was a requirement for use of adjuvant Abemaciclib per Health Canada approval). Other areas of use included guiding the use of chemotherapy (*n* = 7/42, 16.7%), informing prognosis (*n* = 4/42, 9.52%), assessing the response of endocrine therapies (*n* = 4/42, 9.52%), and guiding the use of molecular testing such as Oncotype Dx (*n* = 3/42, 7.14%, Table 2).

### 3.3. Ki-67 Endocrine Responsiveness

Respondents were presented with a brief summary of existing data surrounding the use of Ki-67 endocrine response in adjuvant treatment decision making and asked if they were familiar with the data (Table 3). There were 26/42 (61.9%) respondents who reported being familiar with the data, and 16/42 (38.1%) who were not familiar. Only a small proportion of respondents (*n* = 8/42, 19.0%) reported having previously used short-course pre-operative endocrine therapy for the purpose of assessing endocrine responsiveness. Of those who had used it, six out of eight (75%) had used it for chemotherapy decision making and two out of eight (25%) for other reasons, which they reported as informing whether to use pre-operative ET or whether to use adjuvant Abemaciclib.

When respondents were asked if they would use the Ki-67 endocrine response for prognostication and adjuvant treatment decision making if it was routinely available, 29/42 (69%) responded that they would. For those who reported that they would not use it, reasons included that the data were not robust enough (*n* = 7/13, 53.8%), the approach was too logistically challenging in the current system (*n* = 6/13, 46.2%), they were satisfied with current risk stratification tools (*n* = 5/13, 38.5%), testing was too resource intensive (*n* = 4/13, 30.1%), and other reasons (*n* = 5/13, 38.5%). Most other reasons reported pertained to concerns about reliability and validity of Ki-67 testing.

Respondents were asked what barriers they saw to using Ki-67 endocrine response in practice. The most reported reasons were a lack of timely Medical Oncology consultation prior to surgery (*n* = 22/42, 52.4%), a lack of awareness from other healthcare providers (*n* = 23/42, 54.8%), and an increased healthcare resource requirement (*n* = 23/42, 54.8%). Other reported barriers included potential delays to surgery (*n* = 16/42, 38.1%), modest or unclear benefit (*n* = 8/42, 19.0%), a lack of funding (*n* = 7/42, 16.7%), and an increased risk of toxicity (*n* = 2/42, 4.76%, Table 3). Nine out of forty-two respondents (21.4%) cited other barriers. There were two respondents (4.8%) who did not see any barriers.

### 3.4. Interest in Future Ki-67 Research

**Figure 1:**
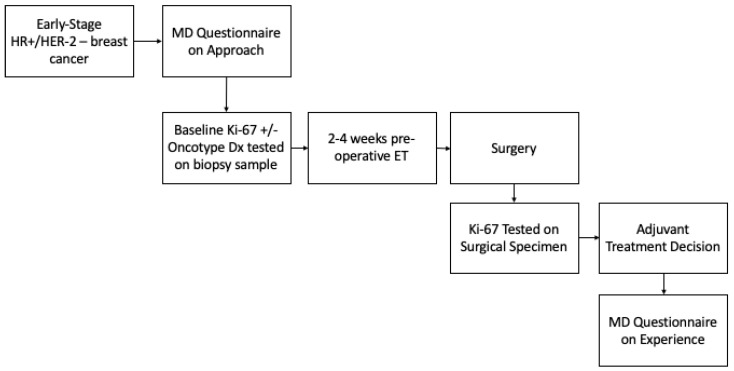
**Proposed study schema.** Proposed future trial utilizing pre-operative endocrine therapy and Ki-67 endocrine response in adjuvant treatment decision making for early-stage hormone-positive breast cancer.

A proposed study involving the use of pre-operative endocrine therapy and Ki-67 endocrine response in adjuvant treatment decision making was outlined for respondents (Figure 1), and they were asked whether they would enroll patients in this study (Table 4). The majority reported that they would (*n* = 35/42, 85.3%). Of the seven out of forty-two (16.7%) who reported they would not enroll patients, five out of seven (83.3%) reported that it was because of logistical challenges in the current practice mode, four out of seven (66.7%) because of lack of funding, one out of seven (16.7%) because of inadequate data to support this approach, and one out of seven (16.7%) for other reasons, which included concerns about over testing and the need for clarity on the potential study. Respondents were also asked what endpoints would be important in such a study. The primary endpoint cited was the number of patients for whom treatment decisions change based on Ki-67 response (*n* = 40/42, 95.2%). Other potential endpoints of interest were a cost analysis (*n* = 18/42, 42.3%), patient-reported satisfaction (*n* = 16/42, 38.1%), and physician satisfaction (*n* = 9/42, 21.4%). Lastly, there were 6/42 respondents (14.3%) who recommended other endpoints, which primarily included efficacy endpoints such as disease-free survival (DFS) and overall survival (OS).

## 4. Discussion

Pre-operative endocrine therapy and the assessment of Ki-67 endocrine response is a treatment strategy endorsed by European guidelines [10]. It allows for assessment of tumor endocrine sensitivity and provides important prognostic information [11,12]. There is also evidence to suggest that the use of Ki-67 endocrine response may allow for a risk-adaptive treatment approach such as that used in the WSG-ADAPT HR+/HER2- trial. This is particularly important, as it provides the oncologist another tool to help guide adjuvant treatment decision making and minimize the risk of overtreatment, especially in the pre-menopausal population, where current molecular testing still recommends consideration of chemotherapy for most patients [4,13]. Despite this evidence, however, it is not a strategy routinely employed in Canada. In fact, North American clinical practice guidelines recommend against its use outside of a clinical trial [18]. To our knowledge, this is the first study exploring physician’s perspectives of, usage of, and perceived facilitators of, and barriers to such a treatment approach in a Canadian landscape.

This survey was devised to gain an understanding of the current use of Ki-67 endocrine response in early-stage hormone-positive breast cancer management in Canada, and to explore barriers to its incorporation into practice. It included breast cancer oncologists from across the various provinces, all with varying location and duration of practice. The majority of respondents reported that Ki-67 testing was available only upon request, not reflexively. The primary reported use of Ki-67 was to guide the use of adjuvant Abemaciclib. This is not surprising given that, at the time of the survey, the Health Canada indication for adjuvant Abemaciclib included having a Ki-67 score of 20% or greater, in addition to specific clinicopathologic features. A minority of providers reported having used short-course pre-operative endocrine therapy for the purpose of assessing endocrine response in order to make adjuvant chemotherapy decisions. This confirms our hypothesis that this is not a regularly used treatment regimen in the Canadian oncology community. Interestingly, almost 40% of respondents were not aware of the data supporting Ki-67 endocrine response, which highlights one contributing reason for why it is not used routinely and should be an area of focus for future continuing education sessions.

Physicians were asked whether they would use Ki-67 endocrine response for prognostication and adjuvant treatment decision making if the information was routinely available. While the majority reported that they would use it, just under one-third of respondents said they would not. One of the primary reasons for why they would not use this approach was that it was felt to be too logistically challenging and resource intensive in our current system. The use of pre-operative treatments such as endocrine therapy in window of opportunity studies has previously been demonstrated to be feasible in a Canadian healthcare system [19]. This, however, does not necessarily translate to it being feasible in standard clinical practice. Studies often have the funding to pay for items such as biopsies or imaging slots, for which timely access can often be difficult in a public and already resource-strained system. Clearly, the feasibility of this approach in a publicly funded healthcare system is an important area for incorporation into future trials using this approach.

The final part of the survey included a proposed study schema, where patients received 2–4 weeks of pre-operative endocrine therapy, and Ki-67 was tested on both the biopsy and surgical specimens in order to test Ki-67 response (Supplementary Material). This proposed study also included surveys on the physicians’ experiences. The overwhelming majority of survey respondents stated that they would enroll patients in such a trial. Endpoints that were felt to be important were the number of patients in which the Ki-67 response changed decision making, followed by cost analysis and patient-reported satisfaction. Interestingly, the reasons for why physicians would not enroll their patients were that it was felt to be logistically challenging in the current practice model, and due to a lack of funding for this regimen. This highlights the need for more pragmatic future trials in this area, and that not only efficacy, but also practicality, are important factors from the physician’s perspective.

A few respondents raised concerns about the strength of Ki-67 as a biomarker. Visual assessment of Ki-67 staining has been shown to have low interobserver concordance, and more so for low and high Ki-67 values [20]. This has, however, been studied by the International Ki-67 Working Group, who has recommended specific operating procedures in order to account for this [21]. There is also intratumor heterogeneity of Ki-67, with levels typically being higher in the periphery of the tumor. Despite these factors, however, the ability of this biomarker to differentiate patient populations is quite well established. Its clinical utility in early breast cancer has been demonstrated by both the POETIC and WSF ADAPT HR+/HER2- trials, and this is what we aim to further explore through trials such as that proposed in our survey [11,12].

Limitations to this study include that it received less responses than desired; however, it is in keeping with response rates from prior physician survey literature such as that by Alzahrani M et al. and McGee S et al., which had response rates of 21% and 42%, respectively [15,17]. We also only surveyed physicians from one country, and the majority of respondents were from one province. Treatment and testing funding algorithms vary largely by province, and thus we may be missing important perspectives from those underrepresented provinces. Additionally, the majority of respondents reported practicing in an academic center. This is likely less of a limitation and more of a representation of the practice landscape for medical oncology in Canada.

## 5. Conclusions

In conclusion, this survey study highlights that, despite evidence supporting its use, Ki-67 endocrine response is not routinely used in Canadian practice. Despite this, there is a high degree of interest in incorporating this approach and participating in future clinical trials that further evaluate this treatment paradigm. Respondents highlighted important barriers, particularly logistical challenges in a publicly funded healthcare system and the need for more robust data to support its efficacy. We aim to design a future pragmatic trial exploring the use of Ki-67 endocrine responsiveness in the adjuvant treatment decision making of early-stage breast cancer.

## Figures and Tables

**Table 1:** **Demographics of survey respondents.** Survey questions exploring the background of respondents, reported as number of responses and percentage of total responses.

Demographics		Number of Respondents	*n* (%)
Prescribe systemic therapy	Yes	48	42 (87.5)
	No		6 (12.5)
Time in independent practice	<5 years	42	7 (16.7)
	5–10 years		10 (23.8)
	10–20 years		13 (30.9)
	>20 years		12 (28.6)
Work Setting	Academic Center	42	37 (88.1)
	Non-Academic Center		5 (11.9)
Province of Practice	Alberta	42	2 (4.76)
	British Columbia		1 (2.38)
	Manitoba		3 (7.14)
	Nova Scotia		3 (7.14)
	Ontario		26 (61.9)
	Quebec		3 (7.14)
	Saskatchewan		4 (9.52)

**Table 2:** **Ki-67 testing usage and availability across Canada.** Survey questions exploring availability and usage of Ki-67 testing, reported as number of responses and percentage of total responses.

Ki-67 Usage Questions		Number of Respondents	*n*(%)
Is Ki-67 testing available for early stage breast cancer at your center?	Yes, performed reflexively	42	1 (2.38)
	Yes, upon request		41 (97.6)
What do you use Ki-67 for in your practice (select all that apply)?	To inform prognosis	42	4 (9.52)
	To guide the use of molecular testing (e.g., Oncotype Dx)		3 (7.14)
	To guide the use of chemotherapy		7 (16.7)
	To guide the use of other adjuvant therapies (e.g., Abemaciclib)		41 (97.6)
	To assess response to endocrine therapies		4 (9.52)

**Table 3:** **Perceptions of, barriers to, and experiences using Ki-67 endocrine responsiveness.** Survey questions exploring usage of Ki-67 in practice and barriers to use, reported as number of responses and percentage of total responses.

Endocrine Responsiveness Questions		Number of Respondents	*n* (%)
Are you familiar with this data?	Yes	42	26 (61.9)
	No		16 (38.1)
Have you used short course pre-operative endocrine therapy for the purpose of assessing endocrine responsiveness before?	Yes	42	8 (19.0)
	No		34 (81.0)
When have you used Ki-67 endocrine response to guide decisions (select all that apply)?	In decision making for chemotherapy	8	6 (75)
	When deciding to order Oncotype Dx		0
	Other		2 (25)
Would you use Ki-67 endocrine response in prognostication and adjuvant treatment decision making if this information was routinely available?	Yes	42	29 (69.0)
	No		13 (31.0)
If no to above, why not?	Data is not robust enough	13	7 (53.8)
	I am satisfied with current risk stratification tools		5 (38.5)
	Testing is too resource intensive		4 (30.1)
	Approach is too logistically challenging with our current system		6 (46.2)
	I do not believe patients will be accepting of this approach		0 (0)
	Other: -Concerns regarding test performance -Would need to review the data further -Unclear how it changes practice		5 (38.5)
What barriers do you see to using this approach (select all that apply)?	Lack of funding for the treatment	42	10 (23.8)
	Potential delay to surgery		16 (38.1)
	Increased healthcare resource requirement		23 (54.8)
	Lack of timely Medical Oncology consultation prior to surgery		23 (54.8)
	Lack of awareness by other healthcare providers		24 (57.1)
	Modest or unclear benefit		8 (19.0)
	Increased risk of toxicity		2 (4.76)
	I don’t see any barriers		2 (4.76)
	Other: -Pathology resources -Access to Ki-67 testing -Need for better data on test performance		9 (21.4)

**Table 4:** **Participant interest in future studies assessing Ki-67 responsiveness.** Survey questions exploring whether respondents would be interested in future clinical trials utilizing endocrine response and which important endpoints to include, reported as number of responses and percentage of total responses.

Future Research Questions		Number of Respondents	*n* (%)
Would you enroll patients in this study?	Yes	42	35 (83.3)
	No		7 (16.7)
If no, please select which apply:	Inadequate data to support this approach	6	1 (16.7)
	Logistical challenges in current practice model		5 (83.3)
	Lack of funding		4 (66.7)
	Other, please specify:		1 (16.7)
What would you see as important endpoints (select all that apply)?	Number of patients where treatment decisions change with Ki-67 response assessment	42	40 (95.2)
	Cost analysis		18 (42.3)
	Patient reported satisfaction		16 (38.1)
	Physician satisfaction		9 (21.4)
	Other: -Efficacy endpoints (disease free survival and overall survival) -Ability to complete concordant ctDNA studies		6 (14.3)

## Data Availability

The data presented in this study are available on request from the corresponding author.

## Supplementary Materials

The following supporting information can be downloaded at: https://www.mdpi.com/article/10.3390/biomedicines12112471/s1, Supplementary Materials: The questionnaire: Use of Ki67 in cancer management in Canada.
