# Supplementary material for: A Multicenter Physician Survey Evaluating the Use of Ki-67 in Breast Cancer Management in Canada
Source: Biomedicines. 2024 Oct 28;12(11):2471. doi: 10.3390/biomedicines12112471 (PMC11592389; doi:10.3390/biomedicines12112471)
Supplement: Supplementary file 1 [file biomedicines-12-02471-s001.zip › biomedicines-3268211-supplementary.pdf]

Supplementary Materials

Use of Ki67 in cancer management in Canada

Physician Questionnaire

Short-course pre-operative endocrine therapy (ET) and measurement of endocrine responsiveness through pre- and post-treatment Ki-67 testing is a strategy employed in the treatment of hormone responsive, early breast cancer (EBC) at centers outside of Canada most predominantly in Europe. This evidence-based approach is supported by multiple randomized, window of opportunity studies, where patients received an intervention e.g., endocrine therapy between the diagnostic biopsy and their planned surgery. The POETIC and WSG-ADAPT HR+/HER-2 – trials are two key studies that demonstrated the benefit of this approach for prognostication and to inform adjuvant treatment decisions in hormone responsive, EBC [1-3-] . This questionnaire is designed to learn about current practices with respect to the use of Ki67, perspectives on data regarding Ki67 as a marker of endocrine responsiveness, and whether there would be interest in a study piloting this approach in Canada.
All responses to this survey will be anonymous. Completion of the survey implies consent to participate. We thank you for your participation.

Assessment of Eligibility:

1. Do you prescribe systemic cancer therapy for patients with breast cancer?

☐ No

☐ Yes

If responded no to this question, please end the survey here.

Demographic Information:

2. How many years have you been in clinical practice?
   - ☐ <5yrs
   - ☐ 5-10yrs
   - ☐ 10-20yrs
   - ☐ >20yrs

3. In what setting do you practice?
   - ☐ At an academic center (teaching hospital)
   - ☐ At a non-academic center (community hospital)
   - ☐ Private practice
   - ☐ Other; please specify: ___________________________________

4. In which province or territory do you practice?
   - ☐ Alberta
   - ☐ British Columbia
   - ☐ Manitoba
   - ☐ New Brunswick
   - ☐ Newfoundland and Labrador
   - ☐ Northwest Territories
   - ☐ Nova Scotia
   - ☐ Nunavut
   - ☐ Ontario
   - ☐ Prince Edward Island
   - ☐ Quebec
   - ☐ Saskatchewan
   - ☐ Yukon
   - ☐ Not applicable

<u>Ki-67 USAGE</u>

5. Is Ki-67 testing available for early-stage breast cancer patients at your center?
   - ☐ Yes, testing is performed reflexively on biopsy and/or surgical specimens
   - ☐ Yes, testing is available upon request
   - ☐ Yes, but testing is available only as part of a study
   - ☐ No, testing is not available

6. If no, are you able to access adjuvant abemaciclib for high-risk patients?
   - ☐ No
   - ☐ Yes

7. What do you use Ki-67 for in your practice? Select all that apply.
   - ☐ To inform prognosis
   - ☐ To guide use of molecular testing (e.g., Oncotype Dx)
   - ☐ To guide used of chemotherapy
   - ☐ To guide use of other adjuvant therapies (e.g., abemaciclib)
   - ☐ To assess response to endocrine therapies
   - ☐ To assess response to non-endocrine therapies
   - ☐ I do not use it

☐ Other; please specify: ________________________________

CURRENT USAGE AND PERCEPTIONS OF ENDOCRINE RESPONSIVNESS IN PRACTICE

Both the POETIC and WSG-ADAPT HR+/HER- trials have shown that Ki-67 response to short-course (2-4 weeks) pre-operative ET is prognostic. In the POETIC trial patients who received short-course pre-operative ET and who's Ki-67 started low and remained low (<10%) had the lowest 5-year risk of recurrence, whereas those whose Ki-67 remained high throughout (did not decrease to <10%), had the highest risk[1].

In the WSG-ADAPT HR+/HER2- trial a patient's endocrine responsiveness (Ki-67 decrease to <10% post short-course pre-operative ET) and Oncotype Dx Recurrence Score (RS) were used to guide adjuvant treatment decisions[2,3]. Those with an RS >25 or 12-25 without endocrine response received adjuvant chemotherapy. They demonstrated that for patients ≤50 years there was no significant difference in 5-year disease free survival (DFS) between those with a RS 0-11 and those with a RS 12-25 plus Ki-67 endocrine response, whereas there was a significant difference between the RS 12-25 plus Ki-67 endocrine response group compared to the non-responders. This supports that pre-menopausal patients who are endocrine responsive can potentially be spared chemotherapy. In the post-menopausal group, there was no significant difference in DFS between RS 0-11 or RS 12-25 plus endocrine response[2,3].

8. Are you familiar with this data?
   ☐ No
   ☐ Yes

9. Have you used short course pre-operative endocrine therapy for the purpose of assessing endocrine responsiveness before?
   ☐ No
   ☐ Yes

10. If yes, when have you used Ki-67 endocrine response to guide decisions? Select all that apply.
    ☐ In decision making for chemotherapy
    ☐ When deciding whether to order Oncotype Dx
    ☐ In other situations. Please specify: ________________________________

11. Would you use Ki-67 endocrine response in prognostication and adjuvant treatment decision making if this information was routinely available?
    ☐ No
    ☐ Yes

12. If no, what is your reason for why you would not use it? Select all that apply.
    ☐ Data is not robust enough
    ☐ I am satisfied with current risk stratification tools
    ☐ Testing is too resource intensive
    ☐ Approach is too logistically challenging with our current system
    ☐ I do not believe patients will be accepting of this approach
    ☐ Other; please specify: ___________________________________

13. What barriers do you see to using this approach? Select all that apply.
    ☐ Lack of funding for the treatment
    ☐ Potential delay to surgery
    ☐ Increased healthcare resource requirement
    ☐ Lack of timely Medical Oncology consultation prior to surgery
    ☐ Lack of awareness by other healthcare providers
    ☐ Modest or unclear benefit
    ☐ Increased risk of toxicity
    ☐ I don't see any barriers
    ☐ Other, please specify: ____________________

FUTURE RESEARCH

We are interested in determining whether an approach similar to that used in the POETIC and WSG-ADAPT trials could be implemented within the Canadian health care system context and would be acceptable for both patients and providers. The ability to perform paired Ki67 testing in window of opportunity studies in Canada has previously been established[4]. We intend to conduct a pilot study where early-stage HR+/HER2- patients receive medical oncology consultation pre-operatively and are offered short course pre-operative endocrine therapy hormonal therapy, with proposed study schema depicted in the figure below. The primary goal of the study will be to assess if Ki-67 response assessment is used by medical oncologists to inform adjuvant treatment decisions.

Proposed Study Schema:

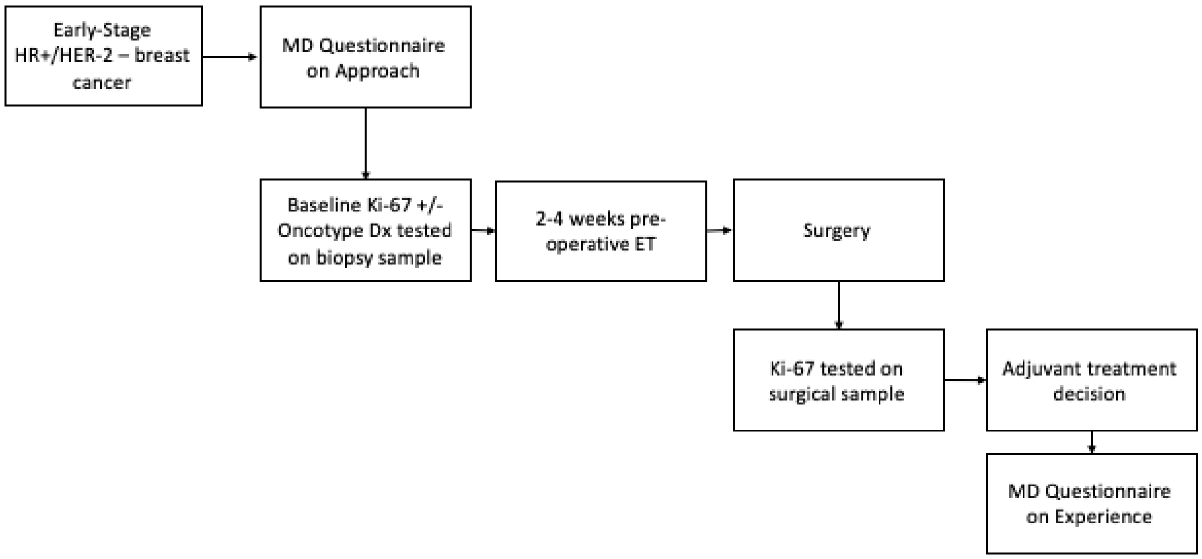

14. Would you enroll patients in this study?
    - ☐ No
    - ☐ Yes

15. If no, please select which apply:
    - ☐ Inadequate data to support this approach
    - ☐ Logistical challenges in current practice model
    - ☐ Lack of funding
    - ☐ Other, please specify: ____________________

16. What would you see as important endpoints (select all that apply)?
    - ☐ Cost analysis
    - ☐ Patient reported satisfaction
    - ☐ Physician satisfaction
    - ☐ Number of patients where treatment decisions change with Ki-67 response assessment
    - ☐ Other, please specify: ____________________

Thank you for completing this survey.
